# The Aggregation Pheromone of *Phyllotreta striolata* (Coleoptera: Chrysomelidae) Revisited

**DOI:** 10.1007/s10886-016-0743-6

**Published:** 2016-08-12

**Authors:** Franziska Beran, Guillermo H. Jiménez-Alemán, Mei-ying Lin, Yun-Che Hsu, Inga Mewis, Ramasamy Srinivasan, Christian Ulrichs, Wilhelm Boland, Bill S. Hansson, Andreas Reinecke

**Affiliations:** 1 Department of Evolutionary Neuroethology, Max Planck Institute for Chemical Ecology, Hans-Knoell-Strasse 8, D-07745 Jena, Germany; 2 Department of Bioorganic Chemistry, Max Planck Institute for Chemical Ecology, Hans-Knoell-Strasse 8, D-07745 Jena, Germany; 3 AVRDC-The World Vegetable Center, Entomology Unit, 60 Yi-min Liao, Shanhua, 74151 Tainan, Taiwan; 4 Department Urban Ecophysiology, Humboldt-Universität zu Berlin, Lentzeallee 55-57, D-14195 Berlin, Germany; 5 Plant Analysis and Stored Product Protection, Institute for Ecological Chemistry, Julius Kühn-Institut, Königin-Luise-Str. 19, D-14195 Berlin, Germany; 6 Behavioural Ecology and Evolutionary Genetics, Max Planck Institute for Ornithology, Eberhard-Gwinner-Str. 7, 82319 Seewiesen, Germany

**Keywords:** Flea beetle, *Phyllotreta striolata*, Aggregation pheromone, Allyl isothiocyanate, Sesquiterpene, (6*R*,7*S*)-himachala-9,11-diene, (3*S*,9*R*,9a*S*)-3-hydroxy-3,5,5,9-tetramethyl-5,6,7,8,9,9a-hexahydro-1H-benzo[7]annulen-2(3H)-one

## Abstract

Aggregations of the striped flea beetle *Phyllotreta striolata* on their crucifer host plants are mediated by volatiles emitted from feeding males. The male-specific sesquiterpene, (6*R*,7*S*)-himachala-9,11-diene (compound A), was shown previously to be physiologically and behaviorally active, but compound A was attractive only when combined with unnaturally high doses of the host plant volatile allyl isothiocyanate (AITC) in field trapping experiments. This indicated that our understanding of the chemical communication in this species is incomplete. Another male-specific sesquiterpenoid, (3*S*,9*R*,9a*S*)-3-hydroxy-3,5,5,9-tetramethyl-5,6,7,8,9,9a-hexahydro-1H-benzo[7]annulen-2(3H)-one (compound G), has been reported from an American *P. striolata* population. We confirmed the presence of compound G, and investigated its interaction with compound A and AITC in a *P. striolata* population in Taiwan. Compound G was attractive to Taiwanese *P. striolata* in laboratory bioassays, but significantly more beetles were attracted to a blend of compounds A and G. Under the same conditions, *P. striolata* showed no preference for the blend of A and G combined with a range of doses of AITC over the sesquiterpenoid blend alone. The sesquiterpenoid blend was tested further in field trapping experiments and attracted significantly more beetles than traps baited with compound A and ecologically relevant amounts of AITC. We conclude that A and G are components of the male-specific aggregation pheromone of *P. striolata* in Taiwan, and that the attractiveness of the pheromone is not reliant on the presence of AITC. Our results further indicate that the male-specific sesquiterpenoid blends differ qualitatively between the Taiwanese and American populations of *P. striolata*.

## Introduction

*Phyllotreta* flea beetles (Coleoptera: Chrysomelidae) aggregate on their host plants, which almost exclusively belong to the order Brassicales. These host plants include many economically important crops such as cabbage, mustard, and canola, and several *Phyllotreta* species are important pests of *Brassica* crops (Andersen et al. 2005, 2006; Lamb 1989). The beetles’ typical “shotgun” feeding damage on cotyledons and leaves can cause considerable crop loss in the seedling stage (Westdal and Romanow 1972) and reduce the marketability of vegetables.

The aggregation behavior of *Phyllotreta* spp. is mediated by volatiles emitted from feeding males (Beran et al. 2011; Peng et al. 1999) and facilitates rapid mass infestations in the field. Comparative headspace analyses from feeding males and females revealed a number of male-specific compounds identified as *γ*-cadinene and himachalene-type sesquiterpenoids (Bartelt et al. 2001, 2011; Beran et al. 2011; Tóth et al. 2012). For example, male *Phyllotreta cruciferae* emit six sesquiterpenoids of which three elicit electrophysiological responses from beetle antennae (Tóth et al. 2005). Field tests showed that (6*R*,7*S*)-himachala-9,11-diene (compound A) is the key aggregation pheromone of *P. cruciferae* (Tóth et al. 2005). The synthetic sesquiterpenoid alone attracted only few adults in the field, but synergistically enhanced the attractiveness of the plant volatile allyl isothiocyanate (AITC), a known attractant for many *Phyllotreta* species (Pivnick et al. 1992; Soroka et al. 2005; Tóth et al. 2005, 2007). Several other *Phyllotreta* species were caught together with *P. cruciferae* (Tóth et al. 2005) suggesting similarities in their chemical communication. Indeed, compound A also was identified as a component of the aggregation pheromone of *Phyllotreta vittula* and *Phyllotreta striolata* (Beran et al. 2011; Tóth et al. 2012). The presence of volatile isothiocyanates (ITCs) was crucial for the behavioral response of *P. vittula* and *P. striolata* to component A in the field (Beran et al. 2011; Tóth et al. 2012). However, the AITC doses required to attract beetles greatly exceeded emission rates from host plants (Najar-Rodriguez et al. 2015; Pivnick and Jarvis 1991) indicating that our understanding of how aggregations occur in these species is still limited.

Isothiocyanates are characteristic defense compounds of plants in the order Brassicales formed via enzymatic hydrolysis of glucosinolates (Halkier and Gershenzon 2006). In intact plant tissue, the corresponding plant enzyme myrosinase, a β-thioglucosidase, is spatially separated from glucosinolates. Herbivore feeding triggers glucosinolate hydrolysis in the damaged plant tissue and non-adapted herbivores are deterred or poisoned by the hydrolysis products, whereas adapted herbivores prevent ITC toxicity using different strategies (Winde and Wittstock 2011). Interestingly, *P. striolata* adults possess an endogenous myrosinase and release small quantities of volatile ITCs derived from glucosinolates they sequestered from their food plants (Beran 2011; Beran et al. 2014). However, these amounts are much less than the doses required for attraction.

We previously identified six male-specific sesquiterpenoid compounds in volatiles from a Taiwanese *P. striolata* population, and found compound A to be physiologically and behaviorally active. Intriguingly, Bartelt et al. (2011) detected a novel male-specific sesquiterpenoid, (3*S*,9*R*,9a*S*)-3-hydroxy-3,5,5,9-tetramethyl-5,6,7,8,9,9a-hexahydro-1H-benzo[7]annulen-2(3H)-one (compound G), as major compound in volatile collections from an American *P. striolata* population. Compound G elicited electrophysiological responses from beetle antennae, but behavioral responses were not assessed. Additionally, (1*S*,2*R*)-2,6,6-trimethylbicyclo[5.4.0]undec-7-en-9-one (compound H) and (6*R*,7*S*)-2,2,6-trimethyl-10-methylene-bicyclo[5.4.0]-undec-1,11-ene (compound I) were detected as minor compound in volatile emissions from American *P. striolata,* but these elicited no electrophysiological activity (Bartelt et al. 2011).

It is assumed that *P. striolata* has been introduced from Eurasia to North America (Bain and LeSage 1998; Smith 1985); however, an analysis of cytochrome oxidase I (COI) revealed 3.3 to 5.7 % sequence divergence between populations from Eurasia and Canada (Beran 2011), indicating that these populations have been separated for at least one million years (Farrell 2001; Juan et al. 1995). With this background, we asked whether population-specific chemical profiles explain the different results obtained in previous studies (Bartelt et al. 2011; Beran et al. 2011). We reassessed the male-specific volatiles from the Taiwanese *P. striolata* population and determined the behavioral responses of *P. striolata* to compounds found, alone and in combination with ecologically relevant amounts of AITC.

## Methods and Materials

### Insects and Plants

*Phyllotreta striolata* adults were collected from crucifer fields at AVRDC-The World Vegetable Center in Shanhua, Taiwan, and shipped to the Max Planck Institute for Chemical Ecology in Jena. The import authorization to Germany was obtained under Directive 2008/61/EC. Adults were maintained in mesh cages (MegaView Science Co., Ltd., Taichung, Taiwan) on potted 3–4-wk.-old *Brassica juncea* cv. Bau Sin plants in a controlled-environment chamber at 24 °C, 65 % relative humidity, and L14:D10 h. Seeds of *B. juncea* were purchased from Known-You Seed Co. LTD, Kaohsiung, Taiwan.

### Volatile Collections

Volatiles were collected from groups of 14 to 20 male *P. striolata* adults feeding on cut leaves of *B. juncea* for one day or for three consecutive days in the laboratory under ambient conditions. Compressed air purified by activated charcoal was passed through a 100 ml glass bottle containing beetles and leaf material at a flow rate of 50–100 ml/min. Volatile compounds were trapped on SuperQ filters (25 mg; ARS Inc. Gainsville, FL, USA), which were afterwards eluted with 100 μl hexane containing 10 ng/μl bromodecane (Sigma-Aldrich) as internal standard. For comparison, collections also were made using activated charcoal filters (1.5 mg CLSA filter, Gränicher & Quartero, Daumazan sur Arize, France), and volatiles were eluted with 30 μl hexane containing 10 ng/μl bromodecane as internal standard (Beran et al. 2011).

### Gas Chromatography-Mass Spectrometry (GC-MS)

Collections of volatiles were analyzed using an Agilent 6890 N gas chromatograph (GC; Waldbronn, Germany) equipped with a ZB-5MSi capillary column (30 m × 0.25 mm ID, 0.25 μm film thickness; Phenomenex, Aschaffenburg, Germany) coupled to an Agilent 5973 quadrupole mass spectrometer (Agilent). The carrier gas was helium at constant flow (1 ml/min). One microliter from each sample was injected in splitless mode into the inlet at 220 °C. The oven program started at 40 °C for 3 min, increased at 10 °C/min to 270 °C, and then with 50 °C/min to 300 °C and held for 2 min. MS conditions were electron impact mode (70 eV), and scan mode 33–250 amu (amu). Male-specific sesquiterpenoids were identified as described in Beran et al. (2011). The identity of compounds G and I detected in volatile collections from *P. striolata* was confirmed by comparing the mass spectra and retention time to reference compounds obtained from Dr. Allard Cossé (USDA-ARS) on the non-polar ZB-5MSi column (conditions as described above) and on an enantiospecific Cyclosil-B column (30 m × 0.25 mm ID, 0.25 μm film thickness, Agilent). The inlet temperature was set to 150 °C to avoid thermal rearrangement of compound G upon injection (Bartelt et al. 2011). The carrier gas was helium at constant flow (1.1 ml/min). The oven program started at 70 °C for 3 min, increased at 1 °C/min to 170 °C, and then at 50 °C/min to 240 °C held for 5 min. MS conditions were electron impact mode (70 eV), and scan mode 33–250 amu.

### Synthesis of Compounds A and G

The synthesis of compound A was performed as described in Jimenez-Aleman et al. (2012), with one modification. We used silica impregnated with CuSO_4_ instead of AgNO_3_ for purification of compound A (Szumilo and Flieger 2001). Due to the higher stability of copper ions relative to silver, CuSO_4_ -modified silica gel can be prepared in advance, handled in the presence of light, stored for several months without appreciable loss of activity and gave separations comparable to those obtained with silica gel impregnated with AgNO_3_.

Plates for thin layer chromatography (TLC) were prepared by dissolving 25 g of CuSO_4_^.^5H_2_O in 100 ml of water and dipping the plate in the solution. TLC plates were dried and activated for 2 h at 120 °C. For column chromatography, 125 g of silica gel were added to a CuSO_4_^.^5H_2_O solution prepared as stated above, the water was evaporated at reduced pressure, and the CuSO_4_ -impregnated silica was activated for 2 h at 120 °C. Compound G was synthesized from compound A as described in Bartelt et al. (2011). The purity of both products A and G was >90 % according to the GC-MS total ion chromatogram (TIC).

### Two-Choice Laboratory Bioassay

Behavioral responses of *P. striolata* adults to the synthetic compounds A and G were determined in a two-choice experiment as described in Beran et al. (2011). Briefly, two traps containing the test compounds or pure solvent applied onto a piece of filter paper were placed in a plastic container with 50 beetles. After 24 h, the number of beetles in each trap was counted. Compound A (5 μg) diluted in hexane and compound G (5 μg) diluted in acetone and dispensed from filter paper were used per trap. To determine the emission of both compounds within 24 h from filter paper, volatile collections were carried out as described above. SuperQ filters were eluted with 200 μl solvent containing 10 ng/μl bromodecane as internal standard.

### Experiment 1

Attraction of beetles to synthetic compound A, synthetic compound G, and compounds A and G combined, were each compared to the corresponding solvent control.

### Experiment 2

Adults were given the choice between compound A and compound G, compound A and the blend of A + G, and compound G and the blend. For Experiments 1 and 2, each combination was replicated 30 times and the position of traps was alternated each time.

### Experiment 3

The interaction of the blend of A + G with AITC (Sigma-Aldrich, Munich, Germany) was investigated by comparing the attractiveness of traps baited with the blend alone and combined with different doses of AITC (10 ng; 100 ng; 1 μg; 10 μg; 100 μg; 1 mg). Each combination was tested 15 times.

### Field Trapping Experiment

A trapping experiment was carried out at AVRDC-The World Vegetable Center in Shanhua, Taiwan (23°07′04.9″N 120°17′42.1″E). Compounds were applied to dental cotton rolls as dispensers (Lohmann & Rauscher International GmbH & Co. KG, Rengsdorf, Germany) and placed in a wing trap (Jackson Traps, Jhen Yong Company, Taiwan). The following treatments were compared: 100 μg of compound A, 100 μg of AITC, 100 μg of compound A and 100 μg of compound G, 100 μg of compound A and 300 μg of compound G, 100 μg of compound A and 300 μg of compound G, and 100 μg of AITC. Dental rolls treated with pure solvent served as control. Traps were placed in a leafy radish field (variety Mei Lu Cai, Taipei Agricultural Products Marketing Co., Taiwan) at a height of 50 cm and arranged in a randomized complete block design with 6 replicates. Blocks were 10 m apart, and the distance between the traps in each block was 5 m. The experiment was performed from 17 to 20 April 2012 and repeated with new randomization in each block and new lures from 24 to 27 April 2012. Beetle numbers in each trap were counted at the end of each experiment. Since significantly more beetles responded to the combination of A and G compared to the individual compounds in laboratory experiments, and supply of synthetic compound G was limited, this compound was not tested individually in the field.

### Data Analysis

Trap count data from two-choice assays were analyzed by Wilcoxon matched-pairs signed-ranks test using the software SigmaPlot version 11. Field trap catches were transformed to log(n + 1) and analyzed by analysis of variance (ANOVA) and *post-hoc* Tukey’s HSD test in SAS Version 9.1.

## Results

### Identification of Compounds G and I in Volatile Collections from *P. striolata* Males

**Fig. 1:**
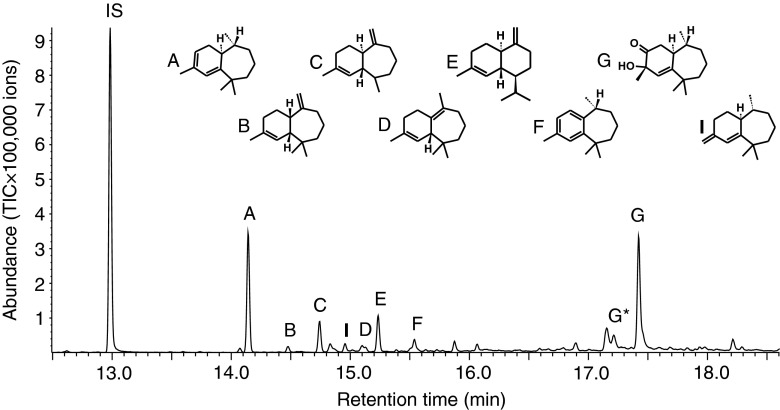
GC-MS analysis (total ion chromatogram) of volatiles collected from feeding male *Phyllotreta striolata* on SuperQ adsorbent. IS, internal standard; A, (6*R*,7*S*)-himachala-9,11-diene); B, *α*-himachalene; C, *trans*-α-himachalene; D, β-himachalene; E, γ*-*cadinene; F, (*R*)-*ar*-himachalene; G, (3*S*,9*R*,9a*S*)-3-hydroxy-3,5,5,9-tetramethyl-5,6,7,8,9,9a-hexahydro-1H-benzo[7]annulen-2(3H)-one; G*, thermal rearrangement product of compound G; I, (6*R*,7*S*)-2,2,6-trimethyl-10-methylene-bicyclo[5.4.0]-undec-1,11-ene

The six sesquiterpenoids (compounds A-F; Table 1) reported previously by Beran et al. (2011) were detected in GC-MS analyses of volatiles collected from male *P. striolata* on SuperQ (Fig. 1). In addition, compound G was detected as a major component in all volatile collections, accounting for ≥50 % of the total sesquiterpenoid blend (Fig. 1; Table 1). The mass spectrum and retention time of compound G were identical to those of a reference compound provided by Dr. Allard Cossé, USDA-ARS, Illinois; USA. The composition of the male-specific sesquiterpenoid blend was similar in 1-d and 3-d volatile collections (Table 1).

**Table 1:** Male specific sesquiterpenoids detected in volatile collections of feeding *Phyllotreta striolata*

Male-specific sesquiterpenoids^a^	Relative abundance (mean % of total ± SD)^b^	
	1 d	3 d
A (6*R*,7S)-Himachala-9,11-diene	23.9 ± 9.5	11.9 ± 5.0
B α-Himachalene	1.8 ± 0.6	1.5 ± 1.3
C *trans*-α-Himachalene	9.7 ± 2.5	8.3 ± 2.2
E γ-Cadinene	11.0 ± 2.4	9.2 ± 3.1
F (*R*)-*ar*-Himachalene	3.8 ± 1.3	4.2 ± 1.4
G (3*S*,9*R*,9a*S*)-3-Hydroxy-3,5,5,9-tetramethyl-5,6,7,8,9,9a-hexahydro-1H-benzo[7]annulen-2(3H)-one	49.6 ± 13.1	65.0 ± 12.1

^a^Minor compounds D and I could not be quantified in samples; means were calculated from 10 (1d) and 9 (3d) volatile collections, respectively

^b^Relative abundance was calculated based on the peak area of single compounds compared to the total peak area of compounds

(6*R*,7*S*)-2,2,6-trimethyl-10-methylene-bicyclo[5.4.0]-undec-1,11-ene (compound I) also was detected by comparison with the mass spectrum and retention time of a reference compound provided by Dr. Allard Cossé using GC-MS. The amounts of compounds D and I were very low, and both compounds often co-eluted with contaminants so they could not be quantified. (1*S*,2*R*)-2,6,6-Trimethylbicyclo[5.4.0]undec-7-en-9-one (compound H) could not be detected in GC-MS analyses of any of the collections by comparison with a reference sample.

**Fig. 2:**
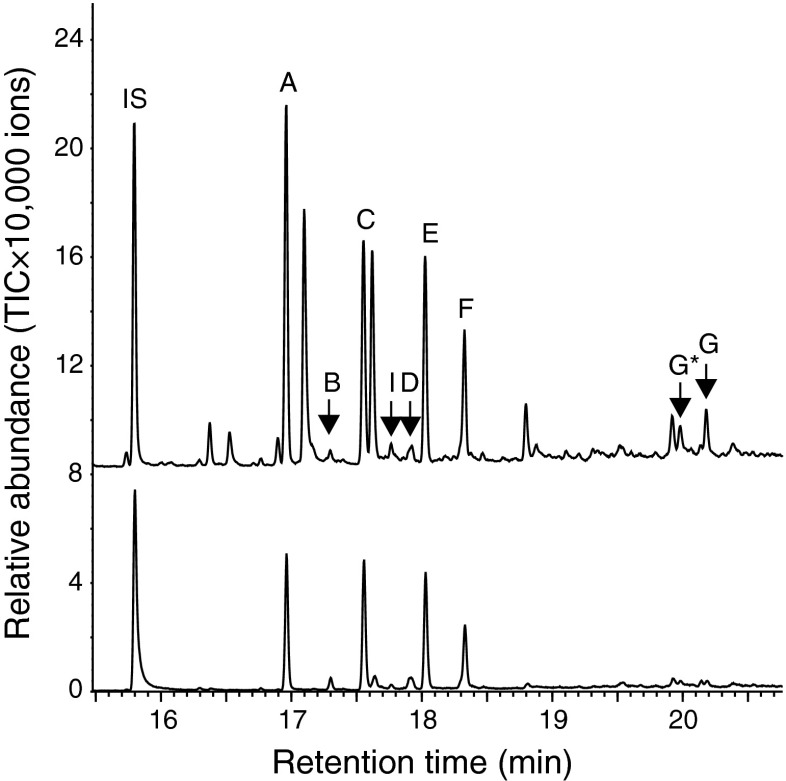
GC-MS analyses (total ion chromatograms) of volatile collections from feeding male *Phyllotreta striolata* using activated charcoal filters. Two example chromatograms are shown to demonstrate the low abundance of compound G. IS, internal standard; A, (6*R*,7*S*)-himachala-9,11-diene; B, *α*-himachalene; C, *trans*-α-himachalene; D, β-himachalene; E, γ-cadinene; F, (*R*)-*ar*-himachalene; G, (3*S*,9*R*,9a*S*)-3-hydroxy-3,5,5,9-tetramethyl-5,6,7,8,9,9a-hexahydro-1H-benzo[7]annulen-2(3H)-one; G*, thermal rearrangement product of compound G; I, (6*R*,7*S*)-2,2,6-trimethyl-10-methylene-bicyclo[5.4.0]-undec-1,11-ene

GC-MS analyses of volatiles from male *P. striolata* collected on activated charcoal showed much lower, often barely detectable, amounts of compound G (Fig. 2).

### Quantification of AITC in Headspace Samples

Allyl glucosinolate is the major glucosinolate of *B. juncea* leaves (Beran et al. 2014), which were used as food in volatile collections with *P. striolata* males. The amount of the glucosinolate hydrolysis product AITC in our samples corresponded to 4.4 ± 1.9 ng (*N* = 10, ±SD) per beetle per day. Whether AITC detected in the sample is released from the plant due to feeding damage or from the beetle cannot be determined in this system.

### Two-Choice Laboratory Bioassay

The amounts of compounds A and G emitted from filter paper over 24 h were 0.57 ± 0.11 μg and 1.49 ± 0.99 μg (*N* = 5; mean ± SD), respectively.

**Fig. 3:**
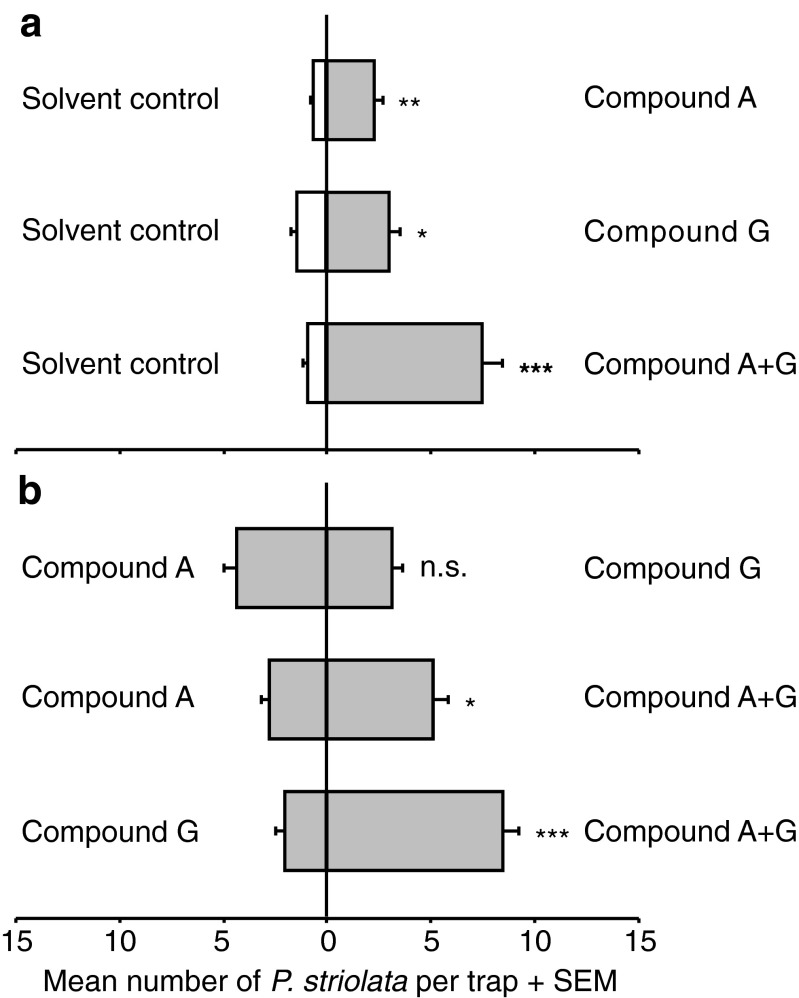
Behavioral responses of male and female *Phyllotreta striolata* to synthetic male-specific sesquiterpenoids in two-choice experiments. **a** Attractiveness of the individual compounds A ((6*R*,7*S*)-himachala-9,11-diene) and G ((3*S*,9*R*,9a*S*)-3-hydroxy-3,5,5,9-tetramethyl-5,6,7,8,9,9a-hexahydro-1H-benzo[7]annulen-2(3H)-one), and the blend of A and G combined compared to the corresponding solvent control. **b** Attractiveness of compound A versus compound G, and the individual compounds versus the blend. Significant differences between the treatments are indicated by * *P* < 0.05; ** *P* < 0.01; *** *P* < 0.001; n.s. *P* > 0.05 (*N* = 30; Wilcoxon matched-pairs signed-ranks test)

**Fig. 4:**
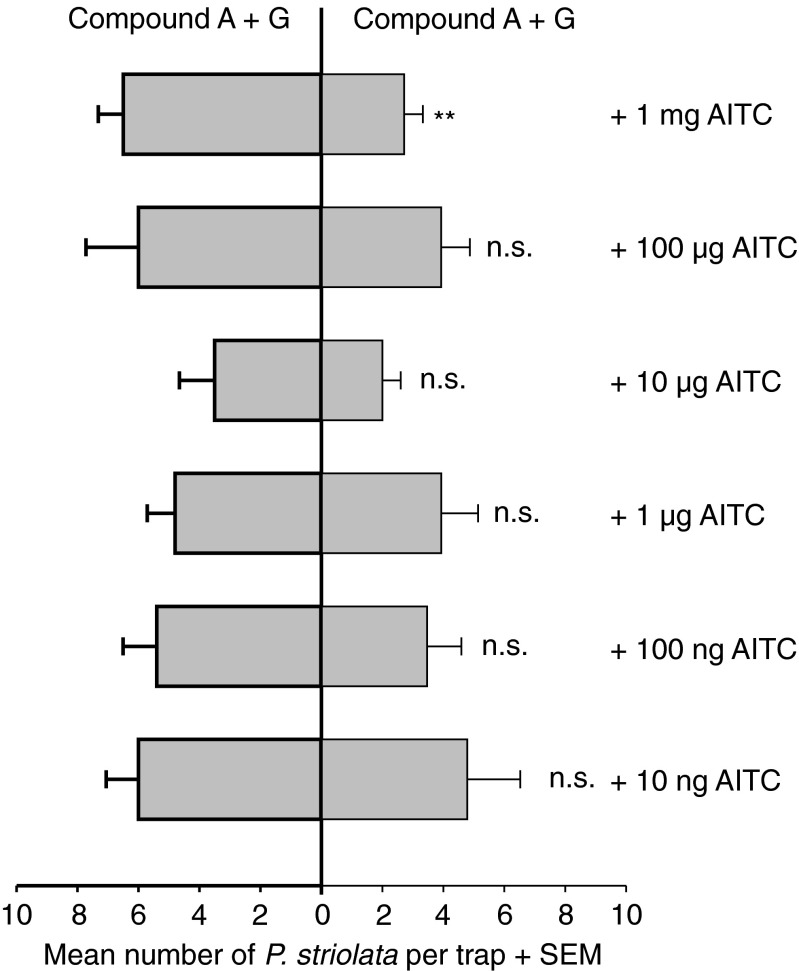
Effect of the glucosinolate hydrolysis product allyl isothiocyanate (AITC) on the attractiveness of the sesquiterpenoid blend to male and female *Phyllotreta striolata* adults in two-choice experiments. Different doses of AITC per trap were tested. Significant differences between the treatments are indicated by ** P < 0.01; n.s. *P* > 0.05 (*N* = 15; Wilcoxon matched-pairs signed-ranks test). A, (6*R*,7*S*)-himachala-9,11-diene; G, (3*S*,9*R*,9a*S*)-3-hydroxy-3,5,5,9-tetramethyl-5,6,7,8,9,9a-hexahydro-1H-benzo[7]annulen-2(3H)-one

In Experiment 1, significantly more beetles were attracted to compound A (*Z* = 2.99, *P* = 0.002), compound G (*Z* = 2.33, *P* = 0.021), and the sesquiterpenoid blend (*Z* = 4.28, *P* < 0.001), compared to the corresponding solvent controls (Fig. 3a). When offered the choice between compound A and compound G in Experiment 2, *P. striolata* responded similarly to both compounds (*Z* = −1.35; *P* = 0.182), but adults clearly preferred the sesquiterpene blend to the single compounds (compound A: *Z* = −2.33; *P* = 0.02; compound G: *Z* = 4.56; *P* < 0.001) (Fig. 3b). In Experiment 3, addition of AITC to the sesquiterpenoid blend had no influence on its attractiveness, except for the highest dose of 1 mg AITC per trap, which attracted significantly fewer beetles than traps baited with the sesquiterpenoid blend alone (*Z* = −2.33; *P* = 0.02) (Fig. 4).

### Field Trapping Experiment

The data from both trapping periods were combined because time (April 17–20 and 24–27) and the interaction treatment × time were not significant (Two-Way ANOVA, time: *F* = 0.08, *P* = 0.783; treatment × time: *F* = 0.72, *P* = 0.634).

**Fig. 5:**
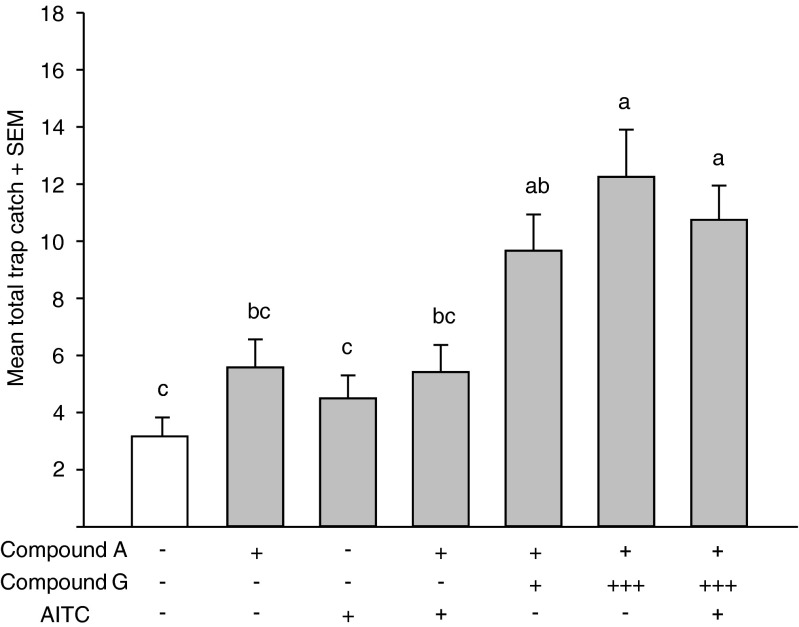
Response of *Phyllotreta striolata* adults to synthetic male-specific sesquiterpenoids and allyl isothiocyanate (AITC) in a field trapping experiment in Taiwan in April 2012. Different letters indicate significant differences between treatments (*N* = 12, ANOVA followed by Tukey’s HSD test; *F* = 8.71, *P <* 0.001). A, (6*R*,7*S*)-himachala-9,11-diene; G, (3*S*,9*R*,9a*S*)-3-hydroxy-3,5,5,9-tetramethyl-5,6,7,8,9,9a-hexahydro-1H-benzo[7]annulen-2(3H)-one; +, 100 μg per trap; +++, 300 μg per trap; −, not present

Traps baited with either 100 μg compound A, 100 μg AITC, or compound A and AITC did not trap significantly more beetles than control traps. A mixture of 100 μg each of compound A and G attracted significantly more beetles than control traps, but these trap catches were not significantly different from traps baited with compound A, or compound A together with AITC. However, traps baited with a high dose (300 μg) of compound G combined with compound A trapped significantly more beetles than compound A with or without AITC (Fig. 5).

## Discussion

Previously, we identified six male-specific sesquiterpenoids (A-F) in volatiles from males of a Taiwanese *P. striolata* population collected on activated charcoal, and of these, compound A was the only physiologically and behaviorally active compound (Beran et al. 2011). Bartelt et al. (2011) identified three additional compounds (G, H, I) in headspace samples from an American *P. striolata* population collected on SuperQ adsorbent, and showed that compound G elicited electrophysiological responses from beetle antennae. In this study we detected compound G as the major male-specific sesquiterpenoid in the headspace of feeding Taiwanese male *P. striolata* collected on SuperQ. Activated charcoal is more retentive than SuperQ and can catalyze rearrangement and/or oxidation of sensitive compounds (Tholl et al. 2006), which most likely explains the low recovery of compound G from activated charcoal using the non-polar solvent *n*-hexane in our initial study.

While traces of compound I were present in volatile collections from males of our Taiwanese population, we could not detect compound H in any of our samples. On the other hand, compounds B and D were not identified in samples from the American *P. striolata* population (Bartelt et al. 2011). These results indicate that the male sesquiterpenoid blends produced by the genetically divergent Taiwanese and North American *P. striolata* populations (Beran 2011) differ qualitatively. However, the physiologically active compounds A and G appear to be conserved. It remains to be determined whether the blend of A and G is behaviorally active in the North American population as well.

Both laboratory assays and field trapping experiments demonstrated that the male-specific compounds A and G mediate attraction in *P. striolata.* In the field, a 1:3 blend of A and G was significantly more attractive than compound A combined with ecologically relevant amounts of AITC. Combining the pheromone mixture with AITC did not increase trap catches in the field, consistent with two-choice laboratory assays, thus demonstrating that the sesquiterpenoid blend could be combined with a wide range of AITC doses without being more attractive than the sesquiterpenoid blend alone. In fact, at the highest dose tested (1 mg AITC per trap), beetles significantly preferred the pheromone blend compared to the blend with AITC. In our earlier study, compound A, the only pheromone component identified at that time, required AITC at supranatural doses to elicit aggregation, thus indicating that other plant volatiles or additional pheromone components mediate aggregation behavior in the field (Beran et al. 2011). The results presented here clarify that the aggregation pheromone emitted by male *P. striolata* consists of at least two components (A and G), and is not reliant on the presence of AITC to attract adult *P. striolata*.

The behavioral response of insects to pheromones may be influenced or even depend on the presence of host plant volatiles (Landolt and Phillips 1997; Reddy and Guerrero 2004; Seybold et al. 2006), and this is likely also the case for the aggregation pheromone of *Phyllotreta* spp. Generally, only few *Phyllotreta* spp. were attracted to synthetic sesquiterpenoids alone in field trapping experiments, whereas high trap catches were achieved when these compounds were combined with ITCs (Soroka et al. 2005; Tóth et al. 2005). In a comparative study, *P. vittula* preferred the combination of pheromone component A and 3-butenyl ITC over the combination with AITC, while significantly more *P. cruciferae* were caught in traps baited with component A and AITC compared to the combination with 3-butenyl ITC (Tóth et al. 2012).

High amounts of ITCs are required to attract *Phyllotreta* spp. For example, AITC release rates of several mg per trap per day were necessary to attract high numbers of *P. striolata* and *P. cruciferae* in the field (Pivnick et al. 1992), but the amounts of AITC detected in volatile collections of *P. striolata* males feeding on leaves of a plant that contains rather high foliar allyl glucosinolate concentrations were relatively low. This was surprising at first but correlates well with *P. striolata* accumulating intact glucosinolates in their bodies (Beran et al. 2014). Apparently, feeding beetles may, at least to certain extent, avoid glucosinolate hydrolysis by the plant myrosinase. The addition of ecologically relevant amounts of AITC to the synthetic sesquiterpenoid blend had no influence on the behavioral response of *P. striolata* adults in laboratory or field experiments. Rather, excessive AITC doses led to a significant preference for the pheromone. This suggests that non-natural stimuli such as individual compounds may be attractive at non-natural concentrations, while ecologically relevant odorant blends may require close to natural ratios and release rates. Together these results indicate that AITC is not an essential cue for host plant location or aggregation because 1) allyl glucosinolate is not generally present in host plants, and 2) the emission rates from traps required for strong attraction of beetles in the field clearly exceed natural emissions rates from individual or small groups of plants (Najar-Rodriguez et al. 2015; Pivnick and Jarvis 1991).

In field experiments presented here and earlier (Beran et al. 2011), catch ratios ranged from about 1:10 to 1:4 for controls compared to the most attractive natural (beetles feeding on plants) and synthetic stimuli, respectively. Trap design and timing of experiments during different field seasons may have contributed to this variation. However, it also is possible that other plant volatiles interact with the pheromone and enhance the behavioral response of *P. striolata* adults to the pheromone. For example, the common monoterpenes (+)-sabinene and (*E*)-β-ocimene, and the green leaf alcohols 1-hexanol and (*Z*)-3-hexen-1-ol were shown to attract *P. cruciferae* adults in an olfactometer (Gruber et al. 2009). We found no attraction of *P. striolata* adults to 1-hexanol or (*Z*)-3-hexen-1-ol in field trapping experiments (Beran 2011), but their potential interaction with the synthetic two-component aggregation pheromone blend has not yet been assessed. Further research is required to establish more comprehensively the role of host plant volatiles in the aggregation behavior of *P. striolata* and other *Phyllotreta* species.
